# Transcriptional regulation of the raffinose family oligosaccharides pathway in *Sorghum bicolor* reveals potential roles in leaf sucrose transport and stem sucrose accumulation

**DOI:** 10.3389/fpls.2022.1062264

**Published:** 2022-12-09

**Authors:** Brian A. McKinley, Manish Thakran, Starla Zemelis-Durfee, Xinyi Huang, Federica Brandizzi, William L. Rooney, Shawn D. Mansfield, John E. Mullet

**Affiliations:** ^1^ Department of Biochemistry and Biophysics, Texas A&M University, College Station, TX, United States; ^2^ MSU-DOE Plant Research Lab, Michigan State University, East Lansing, MI, United States; ^3^ Department of Wood Science, Faculty of Forestry, University of British Columbia, Vancouver, BC, Canada; ^4^ Department of Soil and Crop Sciences, Texas A&M University, College Station, TX, United States

**Keywords:** raffinose, sugar transport, bioenergy sorghum, inositol, phloem loading

## Abstract

Bioenergy sorghum hybrids are being developed with enhanced drought tolerance and high levels of stem sugars. Raffinose family oligosaccharides (RFOs) contribute to plant environmental stress tolerance, sugar storage, transport, and signaling. To better understand the role of RFOs in sorghum, genes involved in *myo*-inositol and RFO metabolism were identified and relative transcript abundance analyzed during development. Genes involved in RFO biosynthesis (*SbMIPS1, SbInsPase, SbGolS1, SbRS*) were more highly expressed in leaves compared to stems and roots, with peak expression early in the morning in leaves. *SbGolS, SbRS, SbAGA1* and *SbAGA2* were also expressed at high levels in the leaf collar and leaf sheath. In leaf blades, genes involved in *myo*-inositol biosynthesis (*SbMIPS1, SbInsPase*) were expressed in bundle sheath cells, whereas genes involved in galactinol and raffinose synthesis (*SbGolS1, SbRS*) were expressed in mesophyll cells. *Furthermore, SbAGA1* and *SbAGA2*, genes that encode neutral-alkaline alpha-galactosidases that hydrolyze raffinose, were differentially expressed in minor vein bundle sheath cells and major vein and mid-rib vascular and xylem parenchyma. This suggests that raffinose synthesized from sucrose and galactinol in mesophyll cells diffuses into vascular bundles where hydrolysis releases sucrose for long distance phloem transport. Increased expression (>20-fold) of *SbAGA1* and *SbAGA2* in stem storage pith parenchyma of sweet sorghum between floral initiation and grain maturity, and higher expression in sweet sorghum compared to grain sorghum, indicates these genes may play a key role in non-structural carbohydrate accumulation in stems.

## Introduction

*Sorghum bicolor* is a drought and heat tolerant C4 grass used throughout the world for production of grain and forage. Bioenergy sorghum is a relatively new sorghum crop that is being selected for high biomass yield, resilience, and a composition optimized for production of biofuels, bioproducts, and biopower (Rooney et al., 2007; Mullet et al., 2014). Bioenergy sorghum exhibits high biomass yield potential due to a long vegetative growing season, high radiation interception efficiency, and good radiation use efficiency due, in part, to C4 photosynthesis (Rooney et al., 2007; Byrt et al., 2011; Mullet et al., 2014). Abiotic stress tolerance is critical for bioenergy sorghum because the crop is targeted for production on annual cropland that is not optimal for food crops due to water limitation and other abiotic stress constraints. Bioenergy sorghum hybrids are more tolerant of drought and heat stress than grain crops because the crop remains in the vegetative phase for most or all of a long growing season thereby avoiding reproductive phase sensitivity to abiotic stress associated with grain crops (Mullet, 2017). In addition, bioenergy sorghum exhibits and is being selected for traits that could further improve adaptation to water-limited environments including but not limited to vertical leaf angles (Truong et al., 2015) and a deep root system (Lamb et al., 2022).

*Myo*-inositol, polyols derived from *myo*-inositol (*i.e.*, pinitol, ononitol), and raffinose family oligosaccharides (RFOs) contribute to the environmental stress tolerance of numerous plants (Valluru and Van den Ende, 2011; ElSayed et al., 2014; Sengupta et al., 2015). For example, the accumulation of high concentrations of raffinose in chloroplasts during heat stress helps protect photosystem II against oxidative damage and contributes to the maintenance of chloroplast membrane integrity (Peters et al., 2007). RFOs have also been implicated in cold acclimation and ROS protection (Nishizawa et al., 2008; Bolouri‐Moghaddam et al., 2010; Lü et al., 2017). In seeds, RFOs are thought to act as osmo-protectants and provide a sugar storage function (Gangl and Tenhaken, 2016; Li et al., 2017). Recently, raffinose and *myo*-inositol have been found to help maintain maize leaf integrity during periods of water deficit (Li et al., 2020).

The initial step in RFO biosynthesis, the synthesis of *myo*-inositol, is catalyzed by D-*myo*-inositol 3-phosphate synthase (MIPS), an enzyme that converts D-glucose-6-P to *myo*-inositol-3-phosphate (Ins3P) (Valluru and Van den Ende, 2011). Ins3P is converted to free *myo*-inositol by inositol mono-phosphatase (InsPase). *Myo*-inositol is used in the synthesis of inositol polyphosphates, phosphatidylinositols, cell wall polysaccharides, and RFOs. Galactinol synthase (GolS), the committed step in RFO biosynthesis, utilizes UDP-galactose and *myo*-inositol to synthesize galactinol (Valluru and Van den Ende, 2011). In poplar, over-expression of *GolS* altered growth and cell wall composition suggesting a signaling role for this cyclitol (Unda et al., 2017). Raffinose synthase (RS) utilizes galactinol to add a galactosyl unit to sucrose thereby producing raffinose, and stachyose synthase (STS) adds an additional galactose to raffinose to generate stachyose. A recent study showed that at high levels of sucrose, ZmRS synthesizes raffinose whereas at low sucrose and high galactinol the enzyme hydrolyzes galactinol generating *myo*-inositol and galactose (Li et al., 2020). In that study, the accumulation of *myo*-inositol or raffinose helped protect leaves from damage during water deficit (Li et al., 2020). Degradation of RFOs is principally carried out by a group of specialized neutral-alkaline α-galactosidases that hydrolyze galactose-galactose and sucrose-galactose bonds present in RFOs (Carmi et al., 2003; Zhao et al., 2006; Peters et al., 2010).

Raffinose family oligosaccharides (RFOs) are involved in sugar storage and transport in numerous plant species. In *Ajuga reptans* raffinose serves a storage function in leaf mesophyll cells and RFO synthesis in vein intermediary cells is loaded into the phloem by polymer trapping for long distance transport (Sprenger and Keller, 2000). In cucumber, a species that transports stachyose, *CsSTS* is expressed in companion cells of the minor veins of mature leaves (Li et al., 2017). This species utilizes both symplastic RFO polymer-trapping and apoplastic sucrose phloem loading strategies (Ma et al., 2019). Similar phloem loading heterogeneity was observed in *Alonso meridionalis* (Voitsekhovskaja et al., 2009). Additional evidence for phloem loading heterogeneity has been reviewed (Slewinski and Braun, 2010; Slewinski et al., 2013).

Sorghum, like maize, is an apoplastic sucrose phloem loading species (Milne et al., 2017). In these C4 grasses, sucrose synthesized in leaf mesophyll and bundle sheath cells diffuses *via* the symplasm from sites of synthesis into vascular bundle parenchyma cells where SWEET transporters unload sucrose into the apoplast followed by SUT1-mediated sucrose transport from the apoplast into companion cell-sieve elements of the phloem for long distance transport (Milne et al., 2017). SUT1 is also expressed in vascular and xylem parenchyma to aid sucrose retrieval from the xylem and vascular apoplast (Baker et al., 2016). Sucrose unloading from the phloem into stem storage parenchyma can occur *via* plasmodesmata (Milne et al., 2017) or through an apoplastic route (Bihmidine et al., 2013) depending on sorghum genotype, stage of internode development and degree of vascular bundle lignification and accumulation of suberin.

Stem sucrose accumulation in sorghum has been the focus of extensive research because ‘sweet sorghum’ genotypes accumulate high levels of stem sucrose during the reproductive phase that are readily extracted and converted to biofuels (Lingle, 1987; HoffmannThoma et al., 1996; Gnansounou and Dauriat, 2010; Gutjahr et al., 2013a; Bihmidine et al., 2015; McKinley et al., 2016; Li et al., 2019). Bioenergy sorghums that accumulate high levels of stem sucrose (~30%) also accumulate starch (up to 12%), glucose and fructose (up to 10% each), and MLG (up to 5%) in stems (Murray et al., 2008; McKinley et al., 2016). Variation in stem carbohydrate accumulation profiles and yield among sorghum genotypes has been traced to differences in phenology, stem morphology, stem juiciness, propensity to form stem aerenchyma (Casto et al., 2018; Xia et al., 2018), genotype by environment interactions, and genetic variation in sugar-signaling and expression of sugar transporters (Murray et al., 2008; Gutjahr et al., 2013b; McKinley et al., 2016). In sorghum, stem carbohydrate levels are low during vegetative phase growth due, in part, to high consumption of UDP-glucose for cell wall biosynthesis (McKinley et al., 2016). Between floral initiation and anthesis, sorghum stem and leaf growth slows and eventually stops, reducing the use of sucrose for growth and cell wall biosynthesis (McKinley et al., 2016). Between floral initiation and anthesis there is a decrease in the expression of genes involved in cell growth and secondary cell wall biosynthesis, and an increase in the expression of genes involved in carbon partitioning and storage in stems (McKinley et al., 2016). Sorghum vacuolar invertase 1 (*SbVIN1*) expression and activity in stems decreases after floral initiation in parallel with accumulation of sucrose in stem cell vacuoles (McKinley et al., 2016). The expression of TST, a sucrose transporter that transports sucrose into the vacuole, also increases during the stem sucrose accumulation phase (Bihmidine et al., 2016). Variation in the extent and kinetics of sucrose accumulation in sorghum stems has been correlated with variation in the level of trehalose-6-phosphate (T6P), a compound involved in sugar-signaling (Li et al., 2019). Therefore, T6P and other sugar-signaling pathways may play a key role in the induction of carbohydrate storage mechanisms in sorghum stems (McKinley et al., 2018).

The potential contribution of *myo*-inositol and RFOs to sorghum resilience and sugar transport has not been investigated. Therefore, in this study information on genes in the sorghum RFO pathway and their expression was characterized in a bioenergy sorghum hybrid (TX08001), a grain sorghum (BTx623) and a sweet sorghum genotype (Della); (i) in leaves, stems, roots, and panicles during plant development, (ii) during a diel cycle in leaves, and (iii) between floral initiation and post-grain maturity when stem sucrose and starch accumulate in stems. The results indicate that genes involved in galactinol and raffinose biosynthesis are differentially expressed in leaf mesophyll cells and that AGAs that hydrolyze raffinose are expressed in leaf bundle sheath and vascular/xylem parenchyma cells of leaf veins, a spatial distribution that may facilitate sucrose retrieval from cells outside of vascular bundles and transport to sites of phloem loading. Post-floral initiation, AGAs were induced >20-fold during the stem sucrose accumulation phase indicating these genes may play a role in the accumulation of non-structural carbohydrates in bioenergy and sweet sorghum stems post-anthesis.

## Materials and methods

### BLASTX and Pfam domain analysis of RS, STS, AGA, and GolS

Sorghum genes involved in RFO metabolism were identified by homology with genes identified in other plant species that encode proteins with validated activity. The homologs from other plants included seven galactinol synthase sequences from *Arabidopsis* (*AtGolS*) (**Table S2**), one raffinose synthase from *Arabidopsis* (*AtRS5*), one raffinose synthase from maize (*ZmRS*), one stachyose synthase (*AtSTS*), and five *Arabidopsis* alpha galactosidases that hydrolyze raffinose (*AtAGA*). Using these sequences as BLASTX queries against the translated protein sequences of the sorghum genome v3.1.1, available through the Phytozome database (https://phytozome.jgi.doe.gov), identified 2 *SbGolS*, 6 *SbAGA*, 1 *SbRS*, and 1 *SbSTS* genes in the sorghum genome that had E-values < 1e^-30^ that were analyzed further. In addition to BLASTX, additional RFO pathway gene family members were identified using sorghum functional annotation key terms and Pfam domains associated with proteins encoded by these gene families. The two SbGolS proteins had a common pfam domain (PF01501.18) which is a conserved domain of the glycosyl transferase 8 family. The SbRS, SbSTS, and the SbAGA proteins shared a common pfam domain (PF05691.11) that is a conserved domain of the glycoside hydrolase family 36. These putative RFO biosynthetic genes are named based on their corresponding maize orthologues (Li et al., 2017).

### Phylogenetic analysis of the RS, STS, and AGA

The evolutionary history of the RS, STS, and AGA genes (excluding GolS) and functionally validated proteins of these families from other species was inferred by using the Maximum Likelihood method and JTT matrix-based model (Jones et al., 1992). Multiple sequence alignment of the 43 protein sequences was performed using the alignment software MUSCLE with default settings implemented in MEGA11. The tree with the highest log likelihood (-36508.47) out of 100 trees is shown. The number of trees in which the associated protein sequences clustered together is shown as a number next to the branches of those sequences. The percentage of trees in which the associated taxa clustered together is shown next to the branches. Initial tree(s) for the heuristic search were obtained automatically by applying Neighbor-Join and BioNJ algorithms to a matrix of pairwise distances estimated using the JTT model, and then selecting the topology with superior log likelihood value. The tree is drawn to scale, with branch lengths measured in the number of substitutions per site. The analysis involved 43 amino acid sequences. There were a total of 1185 positions in the final dataset. Evolutionary analyses were conducted in MEGA11 (Stecher et al., 2020; Tamura et al., 2021). Refer to **Table S1** for a comprehensive list of gene names and gene IDs used in the phylogenetic analysis.

### Enzyme assay

The assay for enzyme activity consisted of a 50 µL aliquot of diluted eluate, 50 µL of 20 mM 4-nitrophenyl-α-D-galactopyranoside (PNPG) (Sigma-Aldrich, St. Louis, MO, USA) and 150 µL of McIlvaine buffer (pH 7.0). The reaction was incubated for 10 min at 37°C, stopped by addition of 1 mL 0.5 M Na_2_CO_3_, and the amount of product formed determined spectrophotometrically using a plate reader (Tecan Infinite^®^ 200 PRO) at 410 nm. The pH optimum of SbAGA1 and SbAGA2 enzymes was determined using McIlvaine buffer (pH from 5.0 to 9.0; in 0.5 pH increments).

### Biochemical analysis of raffinose in sweet sorghums

Plant material for raffinose analysis was obtained from Della grown in a green house in 2011 (McKinley et al., 2016). Tissue was stored in the -80°C long term. Ground tissue was lyophilized in preparation for extraction. Non-structural soluble sugars, including myo-inositol, galactinol, galactose, glucose, sucrose, fructose, raffinose, and stachyose, were extracted from freeze dried leaves and stems. Leaf and stem tissues were first ground in a Thermo Savant FastPrep FP120 Cell Homogenizer (GMI, Ramsey, MN, USA) at 5 m/s for two rounds of 40 s. The microcentrifuge tubes containing dried leaf tissues were briefly dipped in liquid nitrogen to facilitate homogeneous grinding. Once powdered, 35-40 mg of leaf tissue was incubated in 4 mL of methanol-chloroform-water (12:5:3 by volume) at 4 °C overnight, which was subsequently extracted twice with the same solvent mixture and quantified the following day *via* high-performance liquid chromatograph (HPLC), according to the method described by (Park et al., 2009). The residual pellet after soluble sugar extraction was employed for starch analysis, following the protocol of Huang et al. (2020), with amyloglucosidase and amylase digestion prior to HPLC quantification of glucose released by enzymatic digestion.

### Tissue harvest for transcriptome analysis

This study is utilized transcriptome data from several previously published and unpublished experiments. The sorghum lines used in the study included; 1) BTx623, a grain sorghum, 2) Della, a sweet sorghum, and 3) TX08001, an energy sorghum hybrid. The growth and collection of the BTx623 expression atlas plant tissues are described in McCormick et al., 2018. The growth and collection of the plant tissue from BTx623 leaves are described in Murphy et al., 2011. Additionally, a description of the growth and collection of Della plant tissue are described in McKinley et al., 2016. Tissue from the bioenergy sorghum hybrid TX08001 was obtained from plants grown under well-watered, 14-h day greenhouse conditions (29.4°C day/23.9°C night) in 20 cm by 76 cm PVC rhizotrons (Lamb et al., 2022) filled with sandy loam soil and supplemented with 30 g of Osmocote 14-14-14. Plants were thinned to one plant per rhizotron at 10 days after emergence (DAE). Plants were harvested 56, 70, 90, 150, and 170 DAE.

### Template preparation and sequencing

RNA extraction was performed using the Trizol extraction method. The high salt precipitation step was included because of the high concentration of carbohydrates in the sample (https://www.mrcgene.com/rna-isolation/tri-reagent). Total RNA was further purified using the RNeasy kit (Qiagen, Venlo, Netherlands) which isolates RNAs >200 nucleotides in length. An on column DNase1 digestion was performed to remove any contaminating DNA. The quality of the RNA libraries was assessed using an Agilent Bioanalyzer, and the mean RNA integrity number (RIN) from this quality control step was 8.9 and the minimum RIN was 8.3 indicating that the quality of the RNA libraries was high. RNA libraries were prepped for sequencing using the Illumina TruSeq^®^ V2 mRNA Sample Preparation Kit (Illumina, San Diego, CA, U.S.A.), which selects for polyadenylated RNA and excludes rRNA and non-polyadenylated RNAs from the library. The 24 libraries were sequenced on one lane of an Illumina HiSeq 2500 in single end mode.

### RNA-seq analysis of raffinose metabolism genes

The 70 bp reads were aligned to the *Sorghum bicolor* V3.1 genome using the HISAT2 aligner (Daehwan et al., 2015; McCormick et al., 2018). Expression was quantified using the StringTie version 1.3 software (Pertea et al., 2015). Analysis of gene expression was performed on TPM normalized data. Functional analysis of gene families employed gene functional annotations generated by DOE-JGI for the *Sorghum bicolor* V3.1 genome available through Phytozome (https://phytozome.jgi.doe.gov/pz/portal.html) (McCormick et al., 2018).

### Tissue harvest and embedding for *in situ* hybridization

To visualize the location of *SbAGA1*, *SbAGA2*, and *SbRS* transcripts in sweet sorghum tissues, leaf and stem samples were harvested 40 days after grain maturity from Della when the expression of raffinose metabolism genes was expected to be high in Della stems. Plants were grown in a green house in College Station, Texas in the summer of 2018. Leaf and internode samples were harvested from a phytomere located within the center of the functional canopy, which is the area with green, non-senesced leaves attached to the internodes above and below the site of harvest. In both tissues, sections were harvested from the middle of each organ. Tissues were harvested at 11 am, consistent with the harvest time of all the other samples in this study. For fixation, 1 cm wide leaf cross sections and 0.5 cm stem sections were excised from leaves. These sections were vacuum infiltrated with 10% NBF solution and shipped to Michigan State University for further processing. Samples were then washed with 1X PBS and dehydrated through a series of graded ethanol washes, cleared with xylene, and embedded in paraffin wax according to established protocols (Karlgren et al., 2009). Embedded samples were stored at 4°C.

### Probe synthesis and mRNA *in situ* hybridization

RNA probes to perform RNAscope were designed and synthesized by Advanced Cell Diagnostics, Inc. (ACD, Newark, CA, USA) using the custom probe design service. In general, several double Z probe pairs are produced with high specificity hybridization to the target RNA which, when bound in pairs, allows for an amplifier signal to be generated which is then observed as a dot of red chromogenic precipitate. To prepare tissue for staining, embedded sorghum material was thinly sectioned (6µm thick) using a Leica Microtome. Sections were placed on Fisherbrand Superfrost Plus slides (Cat. No. 12-550-15; Thermo Fisher Scientific, Waltham, MA, USA), and placed on a hot plate at 42°C for a few minutes and then further dried overnight at room temperature. Slides were processed as described by (Wang et al., 2012). Specifically, RNAscope 2.5 HD Detection Reagent – RED kit was used (ACD Cat. No. 322360; Thermo Fisher Scientific, Waltham, MA, USA). Slides were sealed with EcoMount (Cat. No. EM897L; Thermo Fisher Scientific, Waltham, MA, USA). Images of plant sections were obtained using bright field through a Zeiss Axio Imager.M2 microscope.

### cDNA cloning of *SbAGA1* and *SbAGA2*

Total RNA was isolated from the mature leaves of field grown Wray sweet sorghum. Samples were harvested from the middle of the leaf pre- and post-anthesis. Leaves were frozen in liquid nitrogen, ground in a mortar and pestle, and extracted using a Tri-Reagent based method with a Direct-zol RNA Miniprep Plus kit (Zymoresearch, Irvine, CA, USA). 5 µg of total RNA was used to synthesize single stranded cDNA using SuperScript IV kit (Invitrogen, Waltham, MA, USA). To prepare recombinant SbAGA1 and SbAGA2 proteins in native form for biochemical characterization, SbAGA1 and SbAGA2 cDNA fragments harboring the full-length coding sequence were PCR amplified with primers: SbAGA1-F: 5’- TTTTGTTTAACTTTAAGAAGGAGATATACATGCACCACCACCACCACCACATGAC-3’, SbAGA1-R: 5’-ATGATGGCTGCTGCCTTCACTGGTACCGAGCTCGT-3’, and SbAGA2-F: 5’-ATTTTGTTTAACTTTAAGAAGGAGATATACATGCACCACCACCACCACCACAT-3’, SbAGA2-R: 5’-ATGATGATGGCTGCTGCCTGGTGTTGTCTTCACTGGTA-3’ containing a 6-His sequence. PCR was performed with Q5 High-Fidelity DNA polymerase (New England Biolabs, Ipswich, MA, USA). The PCR product was gel purified and cloned in-frame into pET28b vector (Millipore Sigma, Burlington, MA, USA) restricted with NcoI (New England Biolabs, Ipswich, MA, USA) enzyme through a Hifi assembly kit (New England Biolabs, Ipswich, MA, USA).

### Expression and purification of recombinant SbAGA1 and SbAGA2

Recombinant *SbAGA1* and *SbAGA2* were expressed in *E. coli* cells, the expression construct was introduced into *E. coli* XJb (DE3) Autolysis™ (Zymoresearch, Irvine, CA, USA) through standard procedures. 100 mL LB broth supplemented with 50 µg/L Kanamycin (Sigma-Aldrich, St. Louis, MO, USA) was seeded with 100 µL of a 2 mL saturated culture grown overnight at 37°C and grown in a 500 mL flask with vigorous shaking. After the culture reached an OD600 of 0.6-0.7, it was induced by addition of 1 mM Isopropyl-β-D-thiogalactopyranoside (IPTG) and further grown for ~24 hours with vigorous shaking at 16°C. Cells were chilled on ice for 30 min before harvesting. All following purification steps were carried out at 4°C.

The culture was harvested by centrifugation at 5,000 g for 10 min and the pellet was stored at -80°C until further use. For purification of recombinant SbAGA1 and SbAGA2, the pellet was thawed and resuspended in 2 mL of 1X Pierce™ 20X Phosphate Buffered Saline (PBS) (Thermo Fisher Scientific, Waltham, MA, USA). The thawed and resuspended cells were lysed using a commercially available lysis buffer supplemented with a Protease Inhibitor Cocktail (Sigma-Aldrich, St. Louis, MO, USA) and the solution was gently shaken for 30 min on ice. After lysis the remaining insoluble residues were removed by centrifugation for 10 min at 15,000 g and the lysate was filtered through a 0.2 µm filter and used for column chromatography.

For purification of His-tagged recombinant SbAGA1 and SbAGA2, the clarified supernatant was applied to 1 mL HisPur™ Ni-NTA spin column (Thermo Fisher Scientific, Waltham, MA, USA) according to manufacturer’s protocol. The column was washed three times with 2 mL of equilibration buffer and bound recombinant protein was eluted with 1.5 mL of elution buffer (50 mM NaH_2_PO_4_, 300 mM NaCl, 1 mM DTT, and 250 mM imidazole and a pH of 8.0). Purified recombinant SbAGA1 and SbAGA2 were analyzed by SDS-PAGE and used for enzyme assays.

### Data analysis and statistics

All data points of this study are the mean of three biological replicates. In graphs that contain error bars, the error bars represent the standard error of the mean.

## Results

### Identification of sorghum genes in the RFO pathway

**Figure 1:**
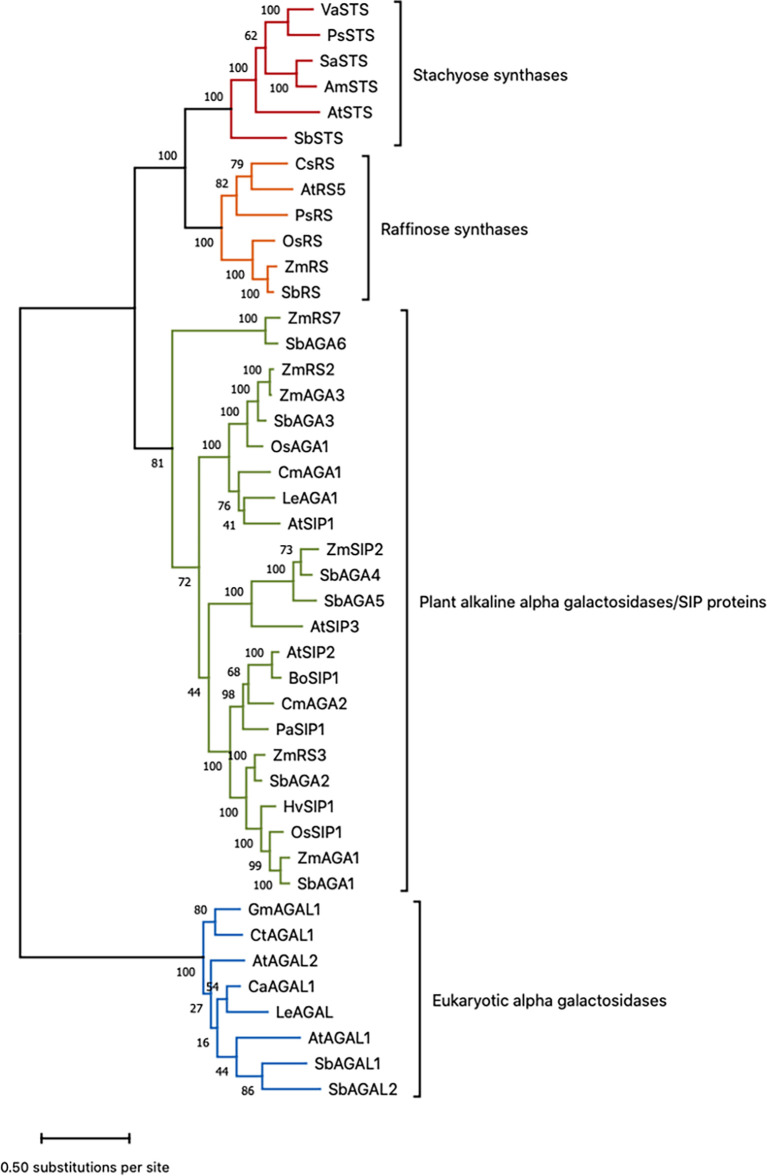
Phylogenetic tree of sorghum proteins and functionally validated RS, STS, and AGA proteins from other species. Refer to **Table S1** for gene name to gene ID conversions. Scale bar shows the number of amino acid substitutions per site. This is the tree with the highest log likelihood out of 100 trees. The number of trees in which the associated protein sequences clustered together is shown as a number next to the branches of those sequences.

Sorghum genes involved in RFO biosynthesis were initially identified based on annotations in the Phytozome database (https://phytozome.jgi.doe.gov/pz/portal.html) (McCormick et al., 2018). The annotated sorghum genes include D-*myo*-inositol 3-phosphate synthase (*MIPS*) (Sobic.001G472800 (*MIPS1*); Sobic.001G261416 (*MIPS2*), inositol monophosphate phosphatase (*InsPase*) (Sobic.009G048900), galactinol synthase (*GolS*) (Sobic.001G391300 (*GolS1*), Sobic.002G423600 (*GolS2*)), raffinose synthase (*RS*) (Sobic.003G052300), and stachyose synthase (*STS*) (Sobic.005G210100). Sorghum genes encoding α-galactosidases/seed imbibition proteins (AGA/SIPs) that are potentially involved in RFO hydrolysis were also tentatively identified through gene annotation. To help confirm the sorghum RFO pathway gene annotations, phylogenetic and protein alignment analyses were carried out using genes with validated *RS*/*STS*/*AGA* activities (Zhao et al., 2006; Peters et al., 2010; Gangl et al., 2015; Li et al., 2017) and the annotated sorghum RFO pathway genes (**Figure 1**, **Table S1**) (McCormick et al., 2018). The results of phylogenetic analysis showed that *SbRS* (Sobic.003G052300) clustered with *ZmRS/OsRS* and that *SbSTS* (Sobic.005G210100) clustered with genes encoding stachyose synthases (STSs) consistent with the gene annotations in Phytozome (**Figure 1**). Six sorghum genes that are annotated as encoding alkaline α-galactosidases (AGAs) clustered with alkaline AGAs encoded by other species (**Figure 1**). The sorghum gene *SbAGA1* (Sobic.002G075800) was most closely related to *ZmAGA1* and *AtSIP2*, genes that encode alkaline AGAs that hydrolyze RFOs (Zhao et al., 2006; Peters et al., 2010). *SbAGA2* clusters with *SbAGA1* in the same subclade of the *AGA* phylogenetic tree (**Figure 1**). *SbAGA3* (Sobic.007G2199003) clusters with *ZmAGA3, OsAGA1, and AtSIP1* (**Figure 1**). *OsAGA1* (Os08g0495800) is involved in degradation of digalactosyl diacylglycerol during leaf senescence (Lee et al., 2009b). *SbAGA4* and *SbAGA5* cluster with *ZmSIP2* and *AtSIP3* (**Figure 1**). *AtSIP3* (= DIN10) is induced during dark treatment by bZIP11 (Valluru and Van den Ende, 2011). Sorghum *SbAGA6* is most closely related to *ZmAGA6*. The *AGA6* genes are diverged from other alkaline AGAs and their substrates have not been identified.

The alkaline AGAs and RFO synthases are related proteins that contain characteristic sequence motifs. Consistent with prior analysis, SbAGA1-6 contain two conserved sequence motifs, DDxW and KxD that are characteristic of AGAs and RS (**Figure S1**) (Carmi et al., 2003). SbRS contains two additional sequence motifs, FM×LGTEA××LG and SGDP×GT×WLQGOHMVHC that distinguish RS from AGAs (**Figure S1**) (Carmi et al., 2003). SbSTS contains an 80 aa motif that distinguishes STS from RF (Gangl et al., 2015).

### Enzymatic activity of SbAGA1 and SbAGA2

To help confirm the predicted functions of SbAGA1 and SbAGA2 the encoded proteins were expressed in *E. coli* and purified with nickel affinity purification. These enzymes were selected for analysis because they exhibit high levels of expression in specific tissues and stages of development (see below) and are homologs of genes previously shown to encode AGAs that hydrolyze RFOs. The expressed proteins were tested for alpha galactosidase activity using the substrate PNPG. **Figure S2** shows the activity of AGA1 and AGA2 across a pH range of 5-9. AGA1 exhibited elevated activity at pH = 7.5 suggesting the protein may be localized in the cytosol. The specificity activity distribution of AGA2 was broader than AGA1 with relatively high activity between pH = 6-7.5.

### Expression of RFO pathway genes in vegetative phase field-grown bioenergy sorghum

**Figure 2:**
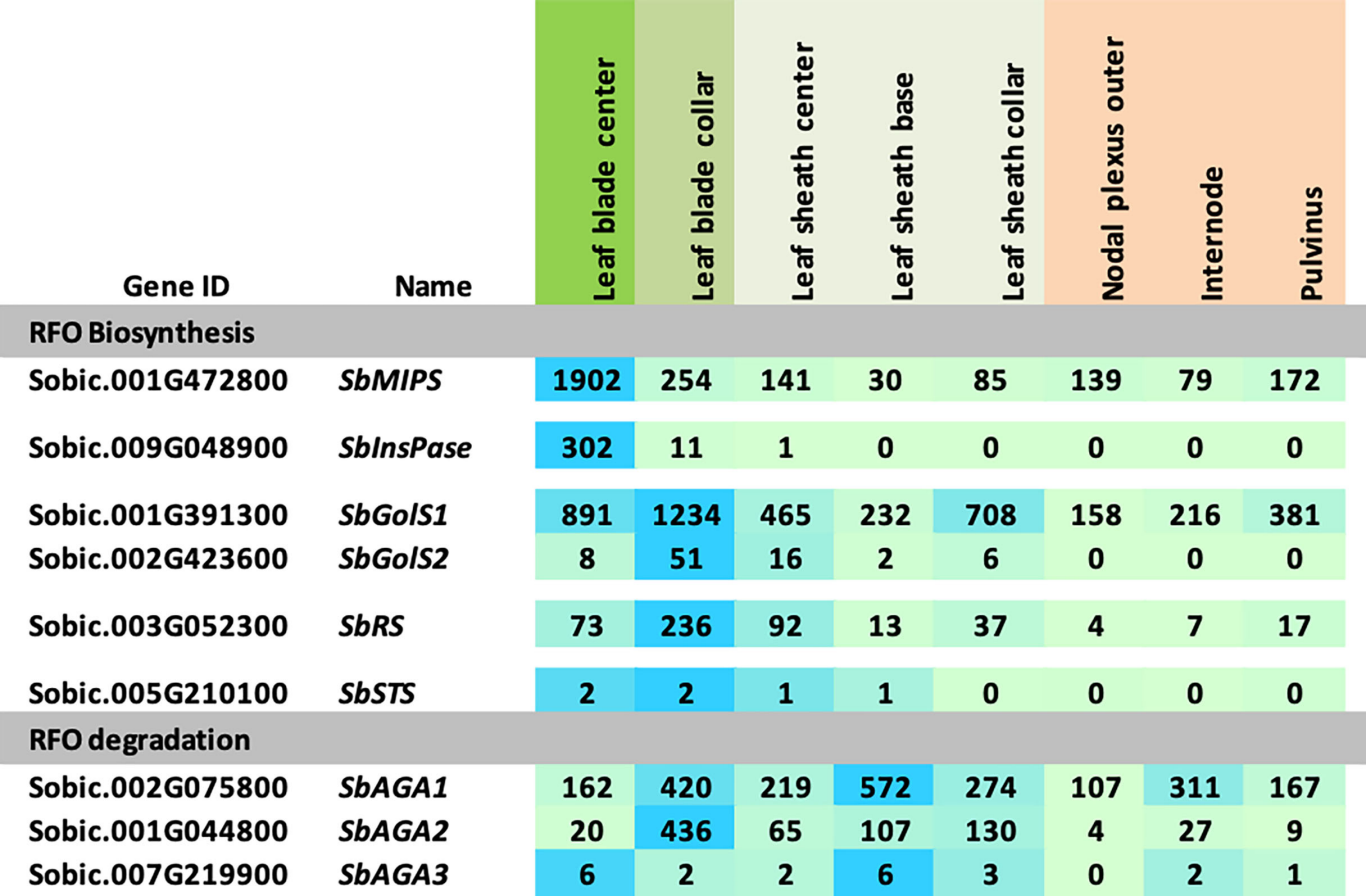
Expression of RFO metabolism genes in various tissues of the energy sorghum hybrid TX08001. Numeric data represents TPM normalized expression. Each data point is the mean of three biological replicates. In the heat map, the darker the blue the higher the expression.

The expression of RFO pathway genes was initially examined in the sorghum bioenergy hybrid TX08001, a genotype that remains in the vegetative phase for >120 days in the field. TX08001 was grown in the field for 60 days (60 DAE) until canopy closure when stems were undergoing rapid elongation. Tissues for transcriptome analysis were collected mid-morning from phytomer (P7) mid-leaf blade, the leaf blade:leaf sheath collar (auricle, ligule), leaf sheath, and tissues that comprise the stem (nodal plexus, internode, pulvinus). RNAseq analysis of RFO pathway expression in these tissues is shown in **Figure 2**. *SbMIPS1* produced four transcripts that varied in abundance in the tissues and organs analyzed. *SbMIPS1* expression was highest in the mid-leaf blade and at lower levels in the leaf collar, leaf sheath and stem tissues. *SbMIPS2* expression was not detected in these tissues. *SbInsPase* was also expressed at high levels in the leaf blade but at very low levels in the leaf collar, leaf sheath and stem. *SbGolS1* was expressed at high levels in the leaf blade, leaf collar and leaf sheath and at lower levels in stem tissues. *SbGolS2* expression was much lower than *SbGolS1*. Expression of *SbGolS2* was highest in the leaf collar and not detectable in the stem. *SbAGA1* mRNA levels were high in most of the tissues analyzed. In contrast, *SbAGA2* was low in the leaf blade, highest in the leaf collar and leaf sheath and low in the stem. *SbAGA3* expression was low in all tissues.

### Expression of RFO pathway genes during grain sorghum development

**Figure 3:**
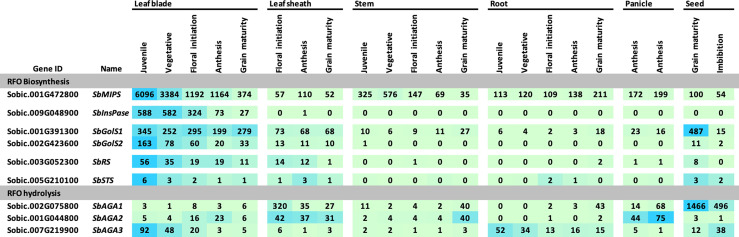
Expression of genes involved in RFO metabolism throughout various tissues and developmental stages of the grain sorghum BTx623. Numeric data represents TPM normalized expression. Each data point is the mean of three biological replicates. In the heat map, the darker the blue the higher the expression.

The expression of sorghum RFO pathway genes during development of the grain sorghum BTx623 was investigated by analysis of transcriptome profiles of leaf blades, leaf sheaths, stems, roots, panicles, and seeds (**Figure 3**) (McCormick et al., 2018). Overall, *SbMIPS1* expression was higher in leaves compared to other tissues during the vegetative phase followed by a gradual decline during the reproductive phase. *SbMIPS2* was not expressed in any of the tissues analyzed except for low expression in the lower panicle at anthesis and therefore was not analyzed further. Expression of *SbInsPase* was high in leaves and very low in leaf sheaths, stems, roots, and panicles throughout development (**Figure 3**). *SbGolS1, SbGolS2, SbRS* and *SbSTS* were also expressed at relatively high levels in leaves and leaf sheaths compared to stems and roots during the vegetative phase, however, the highest expression of *SbGolS1* occurred in seeds at grain maturity (**Figure 3**). *SbAGA* genes showed varied patterns of expression during development (**Figure 3**, RFO hydrolysis). *SbAGA1* and *SbAGA2* were expressed at relatively low levels during vegetative development of leaf blades, stems, and roots. However, expression of these genes was high in the leaf sheath from floral initiation through grain maturity and increased in stems and roots at grain maturity. Both genes were expressed in young developing panicles, but *SbAGA2* expression was low in dry and imbibed seed, whereas *SbAGA1* expression was very high in seeds. *SbAGA3* expression was relatively high in leaf blades and roots of juvenile plants with low expression in leaves, leaf sheaths, and stems during other stages of development.

### Diel cycling of RFO pathway gene expression in grain sorghum leaves

**Figure 4:**
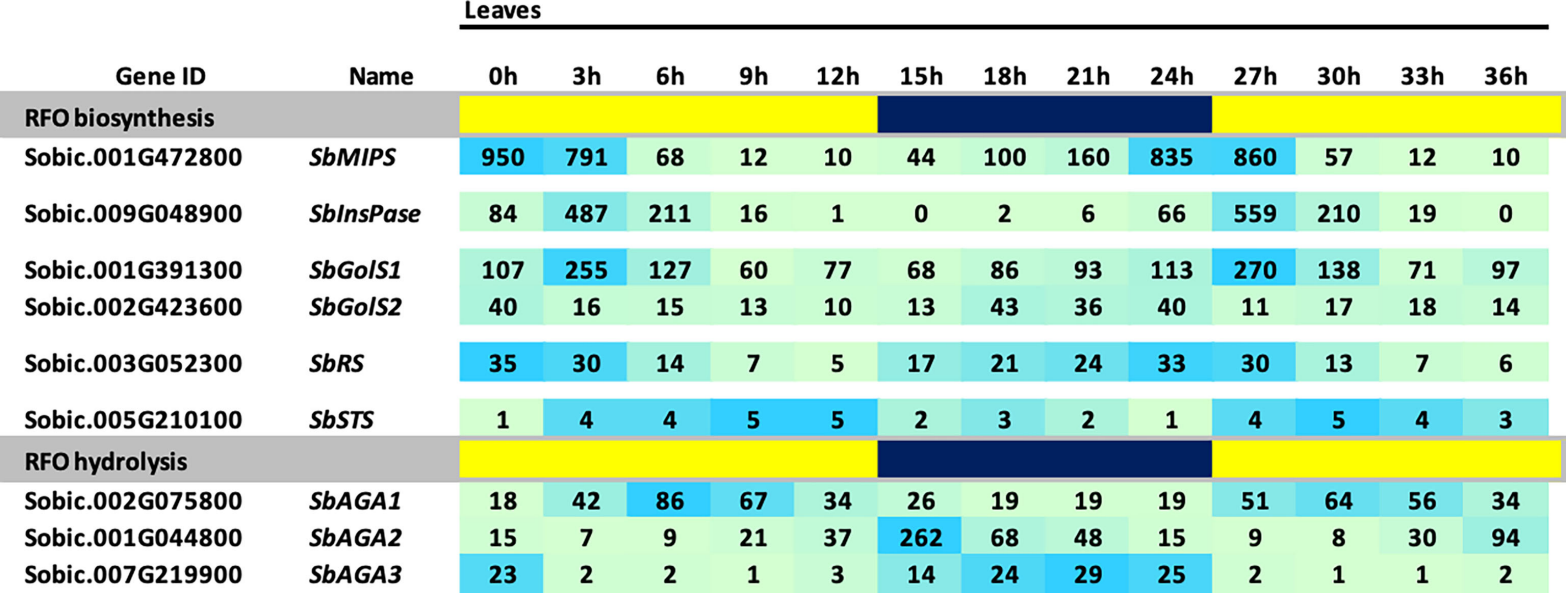
Diel expression of RFO metabolism genes in BTx623 leaves. Yellow highlighted regions indicate lights-on and blue regions indicate lights off. Photoperiod was 14 HL:10HD. Numeric data represents TPM normalized expression. Each data point is the mean of three biological replicates. In the heat map, the darker the blue the higher the expression.

The BTx623 tissues used for RFO pathway expression analysis shown in **Figure 3** were collected ~3 hours after lights on in the morning. Many genes expressed in leaves exhibit diel cycling including genes involved in carbohydrate metabolism that are regulated directly or indirectly by variation in light signaling, photosynthesis and the circadian clock. To determine if the expression of genes in the RFO pathway is regulated during diel cycles in sorghum leaves, RNA-seq profiles were collected from leaves of vegetative phase BTx623 plants every 3 hours during a day-night cycle and the data used to analyze RFO pathway gene expression (**Figure 4**). The analysis showed that expression of genes in the RFO pathway varies significantly during diel cycles (**Figure 4**). *SbMIPS1* and *SbInsPase* mRNA levels increased just before dawn and early in the morning followed by decreasing expression during the day with ~50-fold variation over the diel cycle (**Figure 4**) (Valluru and Van den Ende, 2011). *SbGolS1* and *SbGolS2* were also expressed at higher levels in the morning, but with only ~3-fold variation during the diel cycle. Similarly, *SbRS* expression varied ~3-fold during the diel cycle with higher expression in the morning, a gradual decrease during the day, followed by an increase in expression in the evening and at night (**Figure 4**). In contrast, expression of *SbAGA1* was ~2-fold higher at mid-day (*vs.* night), whereas *SbAGA2* expression was highest in the evening and *SbAGA3* mRNA levels were highest at night. *SbAGA2.2* was the most abundant AGA RNA in leaves in the evening. Overall, *SbAGA2* mRNA levels were ~10-fold higher in leaves for up to 4 hours after lights off in the evening compared to the gene’s RNA level during the morning and most of the day.

### Accumulation of sugars in Della leaves after floral initiation

**Figure 5:**
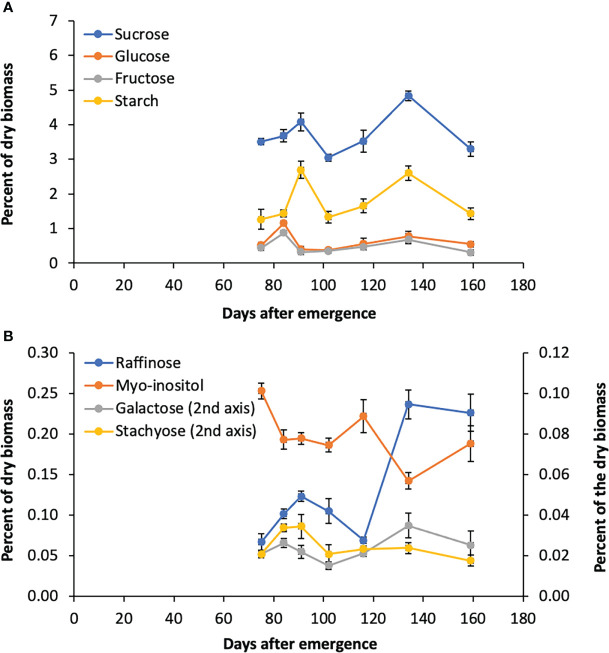
Sugar levels in Della flag leaves from floral initiation to post-grain maturity. **(A)** Levels of sucrose, glucose, fructose, and starch during development. **(B)** Raffinose, myo-inositol, galactose, and stachyose levels during development. Each data point is the mean of three biological replicates. Error bars represent S.E.M.

The accumulation of sugars and starch in stems of Della between floral initiation and post grain maturity was previously quantified, however, data on variation in leaf sugars during this phase of development is lacking (McKinley et al., 2018). To better understand the role of the RFO pathway in leaves, flag leaf sugars were analyzed from tissue collected shortly after floral initiation (75 DAE) through post-grain maturity (159 DAE) (**Figures 5A, B**). Sucrose levels in leaves varied from 3%-4.8% of dry leaf biomass, peaking at anthesis and grain maturity. Starch varied from 1.3% to 2.6% with a pattern of accumulation that was similar to sucrose (**Figure 5A**). The levels of *myo*-inositol and raffinose sugars in leaves was much lower than sucrose, which suggests that raffinose is not being utilized for long term storage (**Figure 5A, B**). It was noted that raffinose levels peak initially at anthesis before declining post anthesis followed by an increase to a maximum between 130-143 DAE when stem sucrose levels are maximal (McKinley et al., 2016).

### RFO pathway gene expression in sweet sorghum leaves and stems post floral initiation

**Figure 6:**
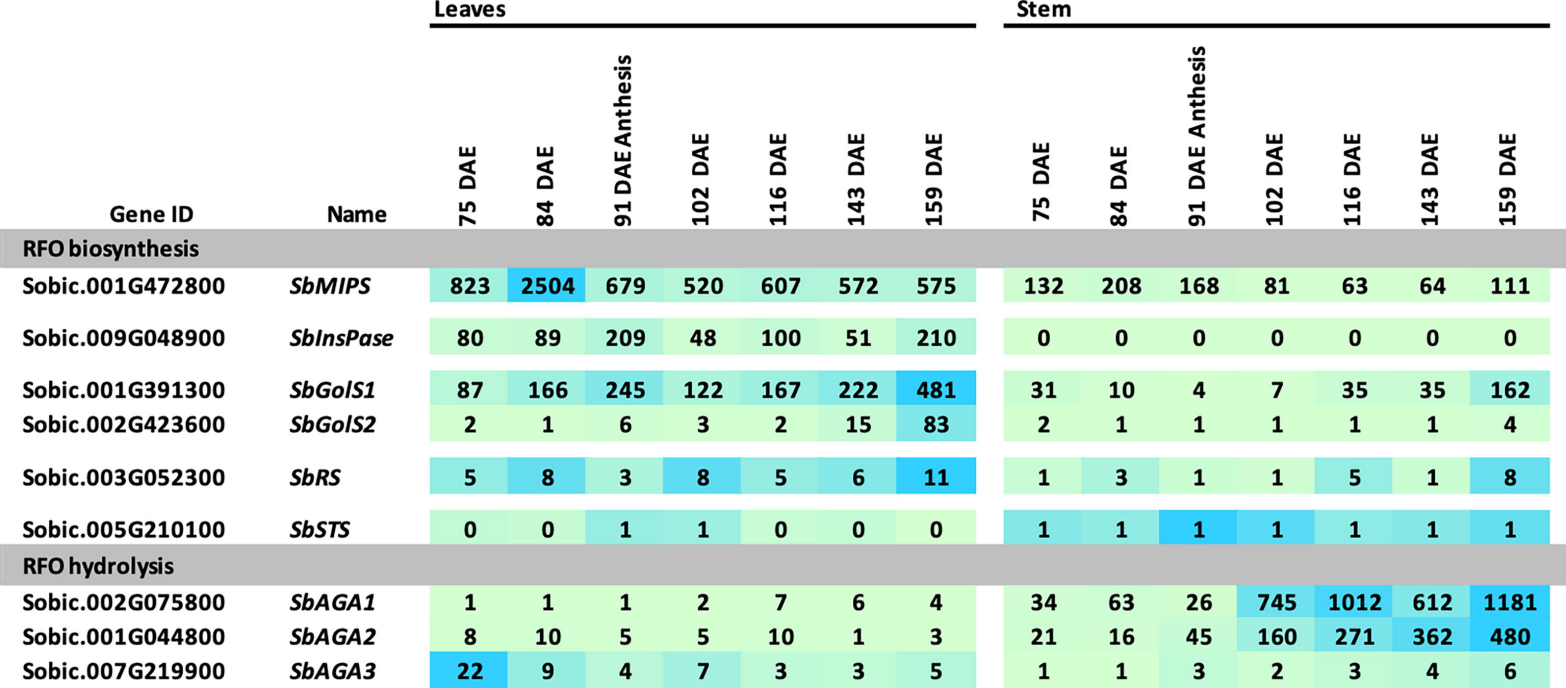
Expression of RFO metabolism genes in leaves and stems of Della during development. Samples were collected starting before anthesis and ending after grain maturity. Anthesis occurred at 91 DAE. Numeric data represents TPM normalized expression. Each data point is the mean of three biological replicates. In the heat map, the darker the blue the higher the expression.

The RFO pathway could potentially play a role in the transport and accumulation of sucrose in sweet sorghum stems. To investigate this possibility, information on RFO pathway expression in leaves and stems was collected between floral initiation (~75 DAE) and post-grain maturity (159 DAE) from Della, a sweet sorghum previously used to investigate the accumulation of sucrose and starch in stems during this phase of development (McKinley et al., 2018). In Della flag leaves, expression of *SbMIPS1*, *SbInsPase*, and *SbGolS1* was maintained at relatively high levels from 75 DAE through post-grain maturity (**Figure 6**). *SbGolS1* RNA levels increased from 75 DAE to a peak at anthesis followed by a decline, then increased to a second peak post-grain maturity (**Figure 6**). *SbGolS2* expression was low in flag leaves until just before gain maturity, and then increased to a peak post-grain maturity (159 DAE). *SbRS* was expressed in flag leaves at a low level from 75-159 DAE. Expression of *SbAGA1-5* was relatively low in flag leaves during this phase of development in Della, although it was noted that leaf tissue was harvested just after dawn when expression of *SbAGA1* is relatively low during the diel cycle in BTx623 leaves (**Figure 4**). *SbAGA6* was expressed continuously in leaves, and *SbAGA3* expression decreased from 75 DAE to 159 DAE.

Expression of genes involved in RFO biosynthesis was generally lower in Della stems compared to leaves (**Figure 6**). *SbMIPS1* mRNA levels were ~2 to 4-fold lower, and *SbInsPase* ~100-fold lower in stems compared to leaves from 75 DAE through grain maturity. Expression of *SbGolS1* in stems decreased post-floral initiation through anthesis, then increased >20-fold post-anthesis to a peak post-grain maturity. In contrast, expression of *SbAGA1* and *SbAGA2* was much higher in stems compared to leaves and increased ~30-fold from early flag leaf stage (75 DAE) to post-grain maturity (159 DAE). RNA levels of *SbAGA4* and *SbAGA5* were low in stems but somewhat higher than in leaves (**Figure 6**). The level of *SbAGA6* mRNA in stems and leaves did not change significantly during this phase of development.

### *In situ* localization of *SbRS, SbAGA1*, and *SbAGA2* RNA in sorghum leaves

**Figure 7:**
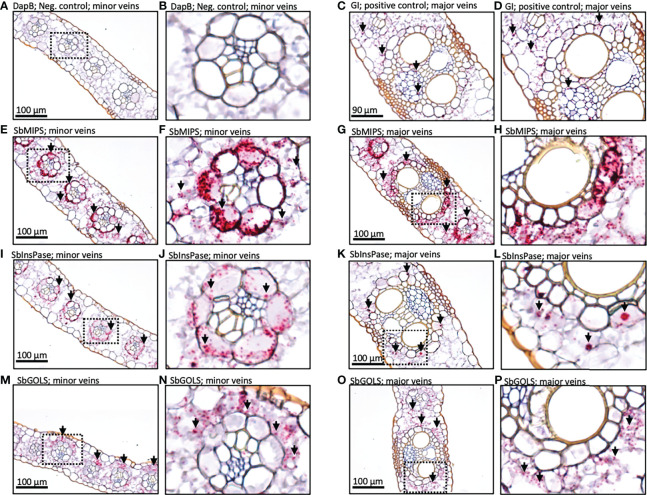
*In situ* localization of *SbMIPS, SbInsPase, and SbGolS1* transcripts in Della leaves. Red punctate structures indicate the location of transcripts. 20X objective. Dashed line boxes in images on the left show where the zoomed in images to the right are focused. **(A, B)** show negative DapB controls in leaves. **(C, D)** show positive Gigantea controls in leaves. **(E & F, I & J, M & N)** show RFO pathway localization of expression in the minor veins of the leaf. **(G & H, K & L, O & P)** show RFO pathway localization of expression in the major veins of the leaf. Red punctate structures indicate the location of transcripts.

**Figure 8:**
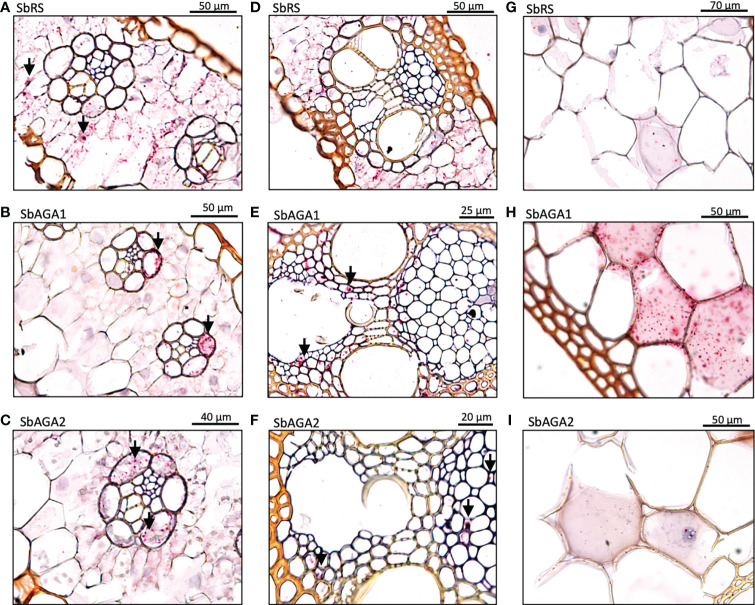
*In situ* localization of *SbRS, SbAGA1*, and *SbAGA2* transcripts in Della leaves. Red punctate structures indicate the location of transcripts. Black arrows represent points of notable localization signals. 40X objective. **(A-C)** show RFO pathway expression in minor veins of the leaf. **(D-F)** show RFO pathway expression in major veins of the leaf. **(G-I)** show RFO pathway expression in parenchyma cells of the midrib of the leaf.

To better understand the role of the RFO pathway in sorghum leaves, the localization of *SbMIPS*, *SbInsPase, SbGolS1*, *SbRS, SbAGA1*, and *SbAGA2* transcripts in Della leaf cells was investigated using the RNAscope *in situ* hybridization platform (Solanki et al., 2020). Fully expanded leaves were collected at 11 am from Della plants 40 days after anthesis (131 DAE). Leaf sections containing minor veins, major veins, and the mid-rib were used in the analysis. Negative controls showed a light pink background in most cells (**Figure 7A, B**), whereas positive controls generated red punctate structures (dots) in cells expressing the target gene (**Figure 7C, D**). In leaves, *SbMIPS1* and *SbInsPase* transcripts were most abundant in bundle sheath cells of minor and major veins with a lower level of transcripts detected in mesophyll cells that surround the bundle sheath (**Figure 7E–H, I–L**). In contrast, *SbGolS1* transcripts were detected principally in mesophyll cells that surround the major and minor veins (**Figure 7M–P**). *SbRS* transcripts were also detected primarily in mesophyll cells of major and minor veins (**Figure 8A, B**), although low levels of transcripts were also detected in the bundle sheath cells of some veins and in midrib parenchyma cells (**Figure 8C**). In addition, in one instance, *SbRS* RNA was high in the vascular parenchyma of a major vein (**Figure S3G**). *SbAGA1* and *SbAGA2* RNA was detected primarily in bundle sheath cells of minor veins (**Figure 8D, G**). In major veins, *SbAGA1* and *SbAGA2* RNA was detected in vascular parenchyma cells and xylem parenchyma cells (**Figure 8E, F, H, I**). *SbAGA1* was also highly expressed in midrib parenchyma cells (**Figure 8F**).

### Differential expression of *SbGolS, SbRS*, and *SbAGA*s in sorghum stems

**Figure 9:**
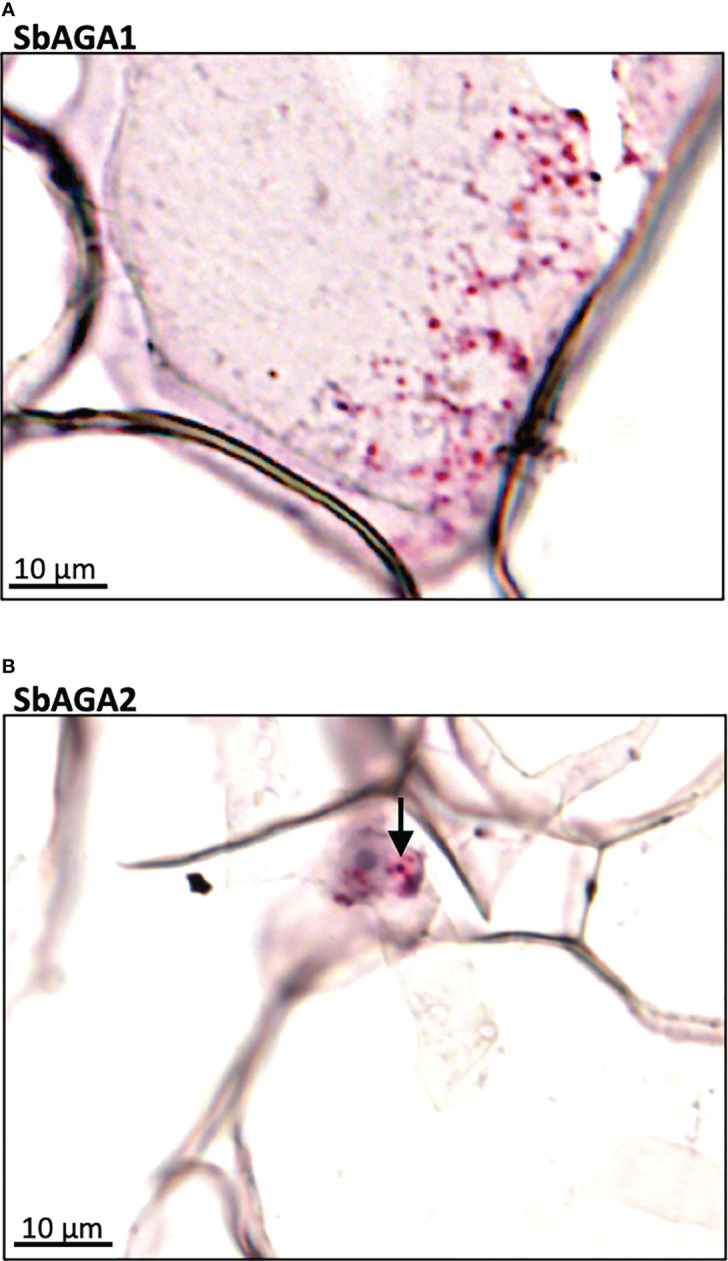
RNAscope data showing the localization of expression of *SbAGA1* and *SbAGA2* in pith parenchyma cells of the stem of the sweet sorghum Della after grain maturity (131 DAE). **(A)** shows the expression of *SbAGA1* and **(B)** shows the expression of *SbAGA2*. The red punctate structures indicate the locations of the transcripts. 40X objective.

Expression of *SbGolS1* and *SbRS* remained high in Della leaves from floral initiation through post-grain maturity. In contrast, transcriptome analysis of Della stems from floral initiation through grain maturity showed that *SbAGA1* and *SbAGA2* expression increased from low levels at floral initiation to very high levels post-anthesis during the sucrose and starch accumulation phase (**Figure 6**). In stems of the grain sorghum BTx623, expression of *SbAGA1* increased from 4 TPM to 40 TPM from floral initiation to post-grain maturity (**Figure 3**) whereas in Della*, SbAGA1* and *SbAGA2* expression increased from 34 TPM and 21 TPM to 1,000 and 470 TPM, respectively during this phase of development (**Figure 6**). *In situ* hybridization analysis showed that *SbAGA1* and *SbAGA2* transcripts were present in ‘storage’ pith parenchyma cells of the stem post-anthesis (**Figure 9A, B**).

### Induction of alpha galactosidases and stem sugars after floral initiation

**Figure 10:**
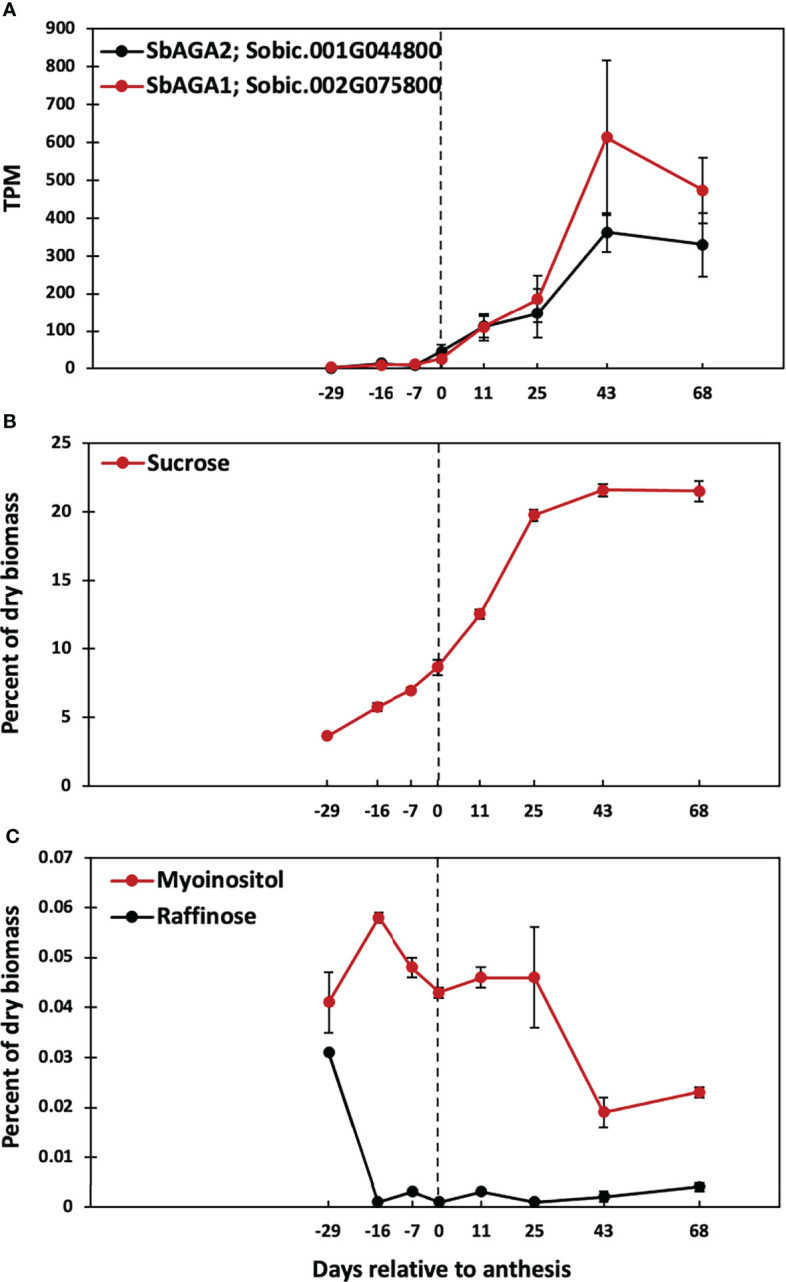
Time course of *SbAGA* expression and sugar accumulation in stems of Della grown in the greenhouse in 2011. Anthesis occurred at 91 DAE and is represented by the dotted line. **(A)** *SbAGA1* and *SbAGA1* TPM normalized expression in Della stems from 62 DAE to 159 DAE. n = 3. Error bars represent S.E.M. **(B)** Internode sucrose content of Della stems from 62 DAE to 159 DAE. n = 3. **(C)** Internode myo-inositol and raffinose content of Della stems from 62 DAE to 159 DAE. n = 3.

A previous study showed that Della sweet sorghum stems accumulate high levels of sucrose and starch between floral initiation and grain maturity (McKinley et al., 2016). Analysis of RNA-seq data from Della stems collected between floral initiation and post-grain maturity revealed that the expression of *SbAGA1* and *SbAGA2* began increasing at anthesis eventually rising to peak expression in stems 43 days post-anthesis (**Figure 10A**). Analysis of stem sugars showed that sucrose levels increase steadily between floral initiation (~75 DAE, -16 days before anthesis) and anthesis (91 DAE) and more rapidly for 25 days post-anthesis (**Figure 10B**, 116 DAE). Raffinose levels in the stem decline to very low levels shortly after floral initiation (between 62 and 75 DAE) and remain low through grain maturity. Stem *myo*-inositol levels increase between floral initiation and 75 DAE then decrease by anthesis (**0,** **Figure 10C**) and further between 116 DAE and 134 DAE (**Figure 10C**).

## Discussion

The overall goal of this study was to characterize the *myo*-inositol-RFO pathway in bioenergy sorghum because of its potential role in drought tolerance, sugar transport and signaling. Sorghum genes encoding steps in the RFO pathway were identified and analyzed using phylogenetic approaches, sequence alignment and motif identification. The analysis confirmed the annotation of *MIPS* and RFO-pathway genes in the BTx623 reference genome (McCormick et al., 2018). The sorghum genome encodes *SbMIPS1*, a gene that produces 4 transcripts of varying abundance, *SbMIPS2*, a gene expressed at very low levels in vegetative tissues, a gene encoding InsPase, RS, and STS, and two genes that encode GolS. In addition, six genes that encode neutral-alkaline alpha-galactosidases were present in the sorghum genome, a class of enzymes previously shown to hydrolyze RFOs (Zhao et al., 2006; Peters et al., 2010). *SbAGA1* and *SbAGA2* clustered with genes that encode alpha-galactosidases that were shown to hydrolyze RFOs in *Arabidopsis* (*AtSIP2*), maize (*ZmAGA1*) and melon (*CmAGA2*). SbAGA1 and SbAGA2 contain protein sequence motifs diagnostic for alpha-galactosidases and lack motifs diagnostic for raffinose synthase (Henikoff et al., 2000; Carmi et al., 2003). In addition, SbAGA1 and SbAGA2 hydrolyzed *p*-nitrophenol-alpha-galactose with pH optima between 6-8 as expected for neutral-alkaline alpha-galactosidases. *OsAGA3*, the closest homolog of *SbAGA3*, encodes an enzyme that hydrolyzes digalactosyl diacylglycerol (DGDG), one of the major glycolipids in chloroplast thylakoids, consistent with a proposed role in chloroplast lipid turnover during leaf senescence (Lee et al., 2004; Lee et al., 2009a). *SbAGA3* expression was higher at night in leaves of BTx623, possibly to support membrane turnover and repair during the night. *SbAGA4* and *SbAGA5* clustered with *AtSIP3* (*DIN10*), a dark-inducible gene associated with leaf senescence that is repressed by sugars (Fujiki et al., 2001) and induced by SnRK1 (Baena-González et al., 2007). *SbAGA6* clustered with *ZmRS7*, genes of unknown function that are diverged from the main cluster of alkaline AGAs.

### RFO pathway expression during development and diel cycling in TX08001 and BTx623

Expression of genes in the sorghum MIPS-RFO pathway was characterized in field-grown TX08001 leaf blades, leaf blade:leaf sheath collar, leaf sheath and stem tissues during the vegetative phase. *SbMIPS1* was highly expressed in leaf blade tissue and at lower levels in the other shoot tissues sampled. *SbInsPase* expression was also high in leaf blades but very low in other shoot tissues an expression pattern also observed in BTx623. In contrast, *SbGolS1* expression was high in leaf blades, even higher in the leaf blade:sheath collar (~1200 TPM) and also expressed in the leaf sheath and stem tissues at >150 TPM. *SbGolS1* expression was significantly higher than *SbGolS2* (~50 TPM), a gene that was selectively expressed in the leaf blade:sheath collar and leaf sheath. *SbRS* expression was highest in the leaf blade:sheath collar (236 TPM) although expression was also observed in leaf blades and sheath indicating a role for raffinose in the leaf blade, collar and sheath tissues. *SbAGA1* expression was relatively high in all of the shoot tissues whereas *SbAGA2* expression was more selectively elevated in the leaf collar:sheath and leaf sheath tissues.

The expression of genes in the sorghum MIPS-RFO pathway was characterized in leaf blades, leaf sheaths, stems, roots, panicles and seeds during development of the grain sorghum BTx623. *SbMIPS1, SbInsPase, and SbGolS1* were expressed at higher levels in BTx623 leaf blades compared to other organs except for high levels of *SbGolS1* expression in seeds at grain maturity. Elevated expression of genes in the MIPS-RFO pathway in leaves indicates that synthesis of *myo*-inositol, galactinol, and raffinose occurs in leaves where glucose-6-phosphate used by MIPS to synthesize *myo*-inositol can be generated from ongoing photosynthesis.

The relative expression of genes in the MIPS-RFO pathway varies extensively in TX08001 and BTx623 in different organs during development. For example, expression of *SbInsPase* was high in leaves but >100-fold lower in leaf sheaths, stems, roots, panicles and seeds, whereas *SbMIPS1* expression was high in leaf blades, but ~3-10-fold lower in other organs. In leaf blades, InsPase can dephosphorylate Ins3P to *myo*-inositol creating substrate for raffinose and phosphatidylinositol biosynthesis. In other organs that have low levels of InsPase, Ins3p could be used directly to produce inositol polyphosphates (Valluru and Van den Ende, 2011).

Additional instances of differential regulation of RFO-pathway genes during organ and plant development in BTx623 were identified. The expression of *SbMIPS* in BTx623 leaf blades was up to 5-fold higher than *SbGolS1* depending on phase of plant development, whereas in the leaf sheath, the expression of these genes was similar. At floral initiation, *SbRS* was expressed in leaf blades and leaf sheaths at similar levels, however, *SbAGA1* and *SbAGA2* RNA levels were up to 10-fold higher in leaf sheaths compared to leaf blades. At floral initiation, *SbMIPS1* expression was similar in roots, leaf sheaths and stems (~100 TPM), but *SbGolS1, SbRS, SbAGA1* and *SbAGA2* expression was very low (0-2 TPM) indicating that only a small portion of the *myo*-inositol synthesized in roots is used for RFO synthesis. In contrast, *SbGolS1* was highly expressed in seeds at grain maturity, ~10-fold higher than *SbMIPS1* expression. This suggests that *myo*-inositol used by GolS1 is derived in part from SbMIPS1 activity during seed development and germination, but also from storage forms of *myo*-inositol such as InsP6 that accumulate in seeds (Raboy, 2003). In maize, galactinol and raffinose accumulate late in seed development (Li et al., 2017; Zhang et al., 2019) and are proposed to provide desiccation tolerance and a source of energy and galactose during germination (de Souza Vidigal et al., 2016).

*SbMIPS* and *SbInsPase* showed ~50-fold variation in expression during a diel cycle with high expression in the morning and very low expression at night. Expression of *SbGolS1* was ~2-fold higher in the morning then decreased in the afternoon, whereas *SbRS* expression increased at night and decreased later in the day (6-12 hours after lights on). *SbAGA1* expression increased during the day peaking between 6-9 hours after lights on, whereas *SbAGA2* and *SbAGA3* expression increased shortly after lights off and then either decreased during the night (*SbAGA2*) or remained high during the night (*SbAGA3*). A large portion of plant genes are regulated during diel cycles (Covington et al., 2008). In many cases, protein levels do not cycle in parallel with changes in RNA, but variation in the amplitude of gene expression during diel cycles are correlated with longer term changes in protein levels (Stitt and Zeeman, 2012). Taken together, the results obtained in the current study indicate that the MIPS-RFO pathway is expressed at higher levels in sorghum leaf blades and that additional regulation of specific genes in the RFO-pathway occurs in different organs. The molecular basis of the observed regulation and its impact on function will be important topics of future research.

### MIPS-RFO pathway gene expression in Della leaves - floral initiation to post grain maturity

*Myo*-inositol and RFO’s are involved in sugar storage, sugar transport and protection from damage associated with abiotic stress in various plants (Santarius, 1973; Stockle and Kiniry, 1990; Bachmann and Keller, 1995; Sprenger and Keller, 2000; Nishizawa-Yokoi et al., 2008; Schneider and Keller, 2009; Valluru and Van den Ende, 2011; Li et al., 2020). The current study examined the potential role of the MIPS-RFO pathway in the synthesis, transport and/or accumulation of sugars in Della, a sweet sorghum that accumulates high levels of stem sucrose post-floral initiation (McKinley et al., 2016; McKinley et al., 2018). Analysis of leaf carbohydrate metabolites in Della leaf blades between floral-initiation and post-grain maturity showed that sucrose, starch and *myo*-inositol levels varied less than 2-fold during this phase of development and that sucrose levels in leaves were >10-fold higher than *myo*-inositol and RFO-sugars. The relatively low levels of *myo*-inositol and RFO-sugars in leaves compared to sucrose/starch indicates RFOs are not serving a significant storage function in sorghum leaves. Sucrose and starch levels in leaves peaked at anthesis, then decreased during grain-filling and peaked again at grain maturity. Raffinose levels also peaked at anthesis, decreased during grain filling, then increased to a higher level at grain maturity. Changes in raffinose levels in leaves were correlated with changes in *SbGolS1* expression, the committed step in raffinose biosynthesis. *Myo*-inositol levels generally decreased from floral initiation through grain maturity. In sugarcane, levels of raffinose in leaves were also correlated with levels of sucrose during diel cycles throughout development (Glassop et al., 2007; De Souza et al., 2018).

While the dynamic changes in MIPS-RFO pathway expression in Della leaves between floral initiation and grain maturity are of interest, the observed variation was not highly correlated with accumulation of stem sucrose and starch post-anthesis. In sorghum, sucrose phosphate synthase (SPS), the enzyme that mediates sucrose biosynthesis, is present in mesophyll (65%) and bundle sheath cells (35%) of sorghum leaves (Lunn and Furbank, 1997). Moreover, it has previously been established that sucrose synthesized in mesophyll cells destined for export moves symplastically to vascular parenchyma cells for SWEET-mediated export into the apoplast followed by SUT1-mediated transport into companion cells-sieve elements for long distance transport. The current study showed that RFOs are not involved in large scale sugar storage in sorghum leaves and only traces of RFOs are present in maize phloem exudates (Yesbergenova-Cuny et al., 2016). What then is the function of the RFO pathway in leaf blades of sorghum plants in the absence of abiotic stress?

**Figure 11:**
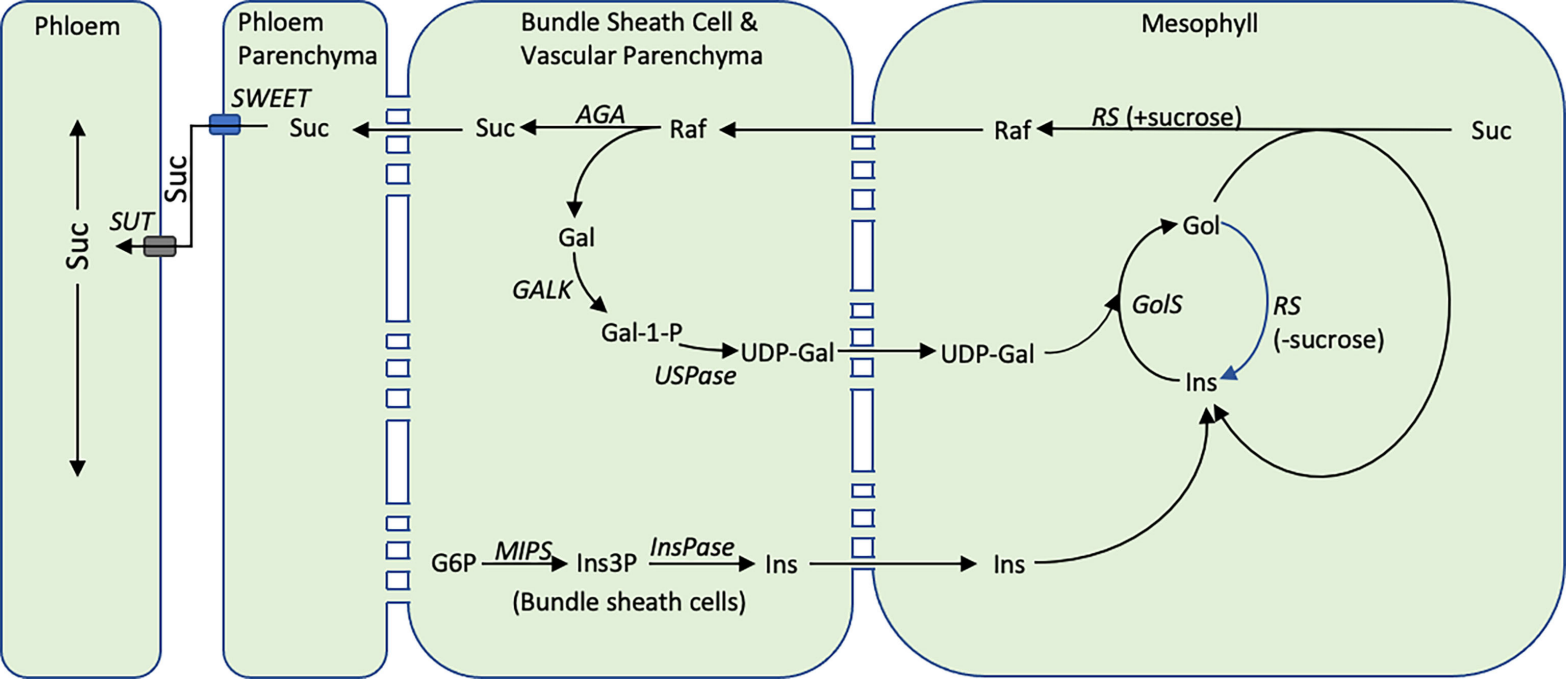
The proposed cycle of RFO biosynthesis in sorghum leaves. Some portion of the sucrose synthesized in the mesophyll cells of sorghum leaves is converted into RFOs. Hydrolysis of RFOs by *AGA1* in bundle sheath cells creates a concentration gradient moving additional sucrose from mesophyll cells into bundle sheath cells thus helping to regulate the concentration of sucrose in mesophyll cells. Hydrolysis of raffinose in bundle sheath cells yields sucrose and galactose. The galactose diffuses back into mesophyll cells for reentry into galactose metabolism. The cellular location of the reactions that convert galactose to UDP-galactose in sorghum are unknown. Inositol synthesis occurs in the bundle sheath cells and inositol potentially diffuses into mesophyll cells where it enters a galactinol metabolic. During the synthesis of RFOs, inositol is released and is recycled. The diagram also shows *RS* being capable of hydrolyzing galactinol to inositol when sucrose concentration is low, while maintaining its normal function, raffinose synthesis, when sucrose concentration is high (Li et al., 2020).

To investigate this question, the expression of *SbMIPS* and RFO-pathway genes in leaf cell-types was investigated using RNAscope technology. The analysis revealed that in leaf blades, *SbMIPS1* and *SbInsPase* were highly expressed in minor vein bundle sheath cells as well as in cells just outside of bundle sheath cells in major veins. *SbGolS1* and *SbRS* transcripts were located primarily in leaf mesophyll cells in minor and major veins. This indicates that *myo*-inositol synthesized in bundle sheath cells, or in cells adjacent to the bundle sheath, could diffuse into mesophyll cells and be used for synthesis of galactinol and raffinose. In contrast, *SbAGA1* and *SbAGA2* were expressed in bundle sheath cells of minor veins and vascular and xylem parenchyma of major veins and the midrib. Therefore, diffusion of RFOs synthesized in mesophyll cells into the bundle sheath of minor veins, or into vascular and xylem parenchyma of major veins/midrib, would result in hydrolysis releasing sucrose and galactose. Sucrose released in vascular parenchyma cells could be unloaded into the apoplast by SWEETs for SUT1-mediated loading into companion cell-sieve elements for long distance transport (Milne et al., 2015). Galactose generated by RFO hydrolysis could diffuse back to leaf mesophyll cells to support continued galactinol biosynthesis (**Figure 11**).

Since sucrose can diffuse to sites of phloem loading in the absence of RFOs, what is the potential added benefit of the proposed RFO-mediated delivery of sucrose from mesophyll cells to vascular parenchyma? The most general explanation could be that the RFO-synthesis-transport-hydrolysis cycle shown in **Figure 11** increases the efficiency of sucrose export from leaf blades. Previously, it has been argued that reducing the sugar and starch content of leaves to a minimum each day increases growth potential by efficiently transporting sugars from sites of synthesis in leaves to growing zones and other major sinks for sugar utilization in vegetative plants (Slewinski et al., 2013). Moreover, accumulation of excess sucrose in leaves is known to trigger feedback mechanisms and an increase in ROS that inhibits photosynthesis (Paul and Foyer, 2001). At high light, when levels of sucrose are high, rates of sucrose unloading from vascular parenchyma by SWEET transporters (Chen et al., 2012) and sucrose loading into companion cells by SUT1 (Slewinski et al., 2009; Baker et al., 2016) are key steps limiting export of sucrose from leaves since mutation of either transporter causes growth defects, abnormal accumulation of sucrose/starch in leaves and leaf senescence. However, at low light, genotypes with non-functional AtSWEET11 and 12 did not show phenotypes (Chen et al., 2012) and maize plants that lack functional SWEET13a/b/c were still capable of setting seed (Bezrutczyk et al., 2018). Moreover, at low light, levels of sucrose in leaves decreases and the concentration gradient of sucrose from outside to inside vascular bundles is expected to diminish (Evert et al., 1978). Under these conditions, movement of raffinose synthesized outside vascular bundles down a steep concentration gradient into vascular parenchyma where hydrolysis takes place may increase sucrose transport into and concentrations in vascular parenchyma maintaining sucrose unloading into the apoplast thus helping to sustain long distance sucrose transport *via* companion cell-sieve elements. It is also possible that sucrose released in vascular and xylem parenchyma could move symplastically into thick walled sieve elements for short distance transport (Botha, 2013).

The proposed cycle of RFO synthesis, transport and hydrolysis could also be involved in the retrieval of sucrose that leaks from vascular bundles and the phloem during long distance transport (Patrick, 1990). In maize, *SUT1* is expressed in companion cells to facilitate phloem loading and long distance sucrose transport, but also in vascular and xylem parenchyma and bundle sheath cells (Baker et al., 2016). Expression of *SUT1* in the latter cells is hypothesized to help retrieve sucrose that leaks from the phloem into the apoplast to maintain high levels of sucrose in vascular parenchyma. Sucrose can leak from vascular bundles into the surrounding tissues *via* the apoplast in the absence of a lignin-suberin barrier or *via* a symplastic route when sucrose levels outside vascular bundles are relatively low. In either case, the RFO synthesis from sucrose in cells outside the vascular bundle, diffusion and hydrolysis in vascular/xylem parenchyma would provide a mechanism for retrieving sucrose that leaks from vascular bundles and transporting it back into vascular bundles for long distance transport. In this regard, we noted that RFO-pathway genes are expressed in the leaf collar and leaf sheath and that expression of *SbAGA1* and *SbAGA2* in the leaf sheath was generally higher than in leaf blades. The leaf collar and sheath are more shaded than the leaf blade and not a primary source of photosynthate, however, significant leakage of sucrose could occur from leaf sheath vascular bundles. It is possible that RFO synthesis, transport and AGA mediated hydrolysis in vascular parenchyma could help retrieve sucrose in the leaf sheath.

### Potential role of the RFO pathway in stem sucrose accumulation

In Della, *SbAGA1* and *SbAGA2* expression increased >20-fold in stems between anthesis and grain maturity reaching expression levels of ~1,000 TPM (*SbAGA1*) and ~470 TPM (*SbAGA2*), respectively by grain maturity. *In situ* analysis showed that *SbAGA1* and *SbAGA2* transcripts were present in stem pith parenchyma cells of Della stems post-anthesis, suggesting that AGA1/2 may play a role in sucrose accumulation in sweet sorghum stems. A significant increase in *SbAGA1* and *SbAGA2* expression between the vegetative phase and grain maturity was also observed in the stems of the sweet sorghum Wray. In the grain sorghum BTx623, expression of *SbAGA1* and *SbAG2* also increased in stems between floral initiation and grain maturity, but maximum expression was much lower (~40 TPM).

The increase in expression in *SbAGA1* and *SbAGA2* started at anthesis and reached a maximum at, or shortly after, grain maturity. Stem sucrose levels increase after floral initiation before the increase in *SbAGA1/2* expression and reach ~30% of maximum at anthesis in parallel with a decrease in stem/leaf growth, reduced expression of *SbVIN1* and genes involved in secondary cell wall formation (McKinley et al., 2016). Sucrose continued to accumulate in stems post anthesis to 95% of maximum about 10 days before grain maturity. *SbAGA1/2* expression in Della stems peaked at grain maturity. In maize, treatment of embryonic callus cells with ~5% sucrose induced *ZmAGA1* transcript levels (Zhao et al., 2006). Induction of *SbAGA1* expression in stems by high levels of sucrose is consistent with the observed increase in *SbAGA1* mRNA levels following increases in stem sucrose.

*AGA1/2* expression is high in many sucrose accumulating tissues. For example, *CmAGA1* and *CmAGA2* are highly expressed in the sucrose accumulating melon fruit mesocarp (Gao et al., 1999; Carmi et al., 2003). In *Arabidopsis*, *AtSIP2*, a homolog of *SbAGA1*, is differentially expressed in young leaves that are importing sucrose and in vascular non-xylem tissue of roots that is transporting sucrose (Peters et al., 2010). In RFO-transporting species such as melon or cucumber, AGA1 is proposed to facilitate RFO unloading into sink tissues by hydrolyzing raffinose and stachyose that is transported from leaves to sink tissues releasing sucrose for further metabolism. However, in C4 grasses such as maize, only trace levels of RFOs have been detected in phloem exudates (Ohshima et al., 1990; Yesbergenova-Cuny et al., 2016) therefore AGA is not required to maintain the flow of RFO-derived sugars into sink tissues of these species. In sweet sorghum stems, sucrose is unloaded from the phloem *via* an apoplastic route (Bihmidine et al., 2015) or a symplastic route (Milne et al., 2015) into surrounding pith parenchyma for storage in the large vacuoles of these cells. We hypothesize that high expression of *SbAGA1* and *SbAGA2* may function in RFO-mediated sucrose retrieval from stem cells not involved in sucrose/starch storage by the mechanism proposed to operate in leaves except that RFO hydrolysis releases sucrose in stem pith cells instead of leaf vascular parenchyma cells. The expression of *SbAGA1* in leaf vascular parenchyma and in stem pith parenchyma that are accumulating sucrose is consistent with induction of *AGA1* genes in cells that accumulate high levels of sucrose. *SbAGA1* was also highly expressed in sorghum seeds, another strong sink for sucrose that may benefit from the proposed RFO-mediated sucrose retrieval pathway.

## Data availability statement

Data from this study are hosted by the JGI Genome Portal https://genome.jgi.doe.gov/portal. Below are the JGI project names and the JGI project IDs that can be used to search for the datasets. 1. Sorghum bicolor Della internode development, JGI Project ID: 1190869. 2. Sorghum bicolor Della leaves development, JGI Project ID: 1240803. 3. Sorghum bicolor Circadian Cycling gene expression profiling, JGI Project ID: 1051031. 4. Sorghum bicolor BTx623 Gene Atlas (plate 8, 9, and 10), JGI Project IDs: 1051038, 1051407, 1053826.

## Author contributions

BM, MT and JM conceived the original research plans. BM and MT performed most of the experiments and analyzed the data. SZ-D performed RNA *in situ* hybridization experiments. XH conducted HPLC analysis of biomass. SM, FB, and JM supervised the experiments. BM and JM wrote the manuscript. All authors contributed to the article and approved the submitted version.

## Supplementary material

Supplementary Figure 1 Sequence analysis of sorghum proteins with functionally validated RS, STS, and AGA protein sequences from other species. Sequence alignments between RS and AGA proteins show sequence motifs in common [DDxW, KxD], and sequence motifs that help to differentiate RS from AGAs [FMxLGTEAxxLG, SGDPxGTxWLQGOHMVHC].

Supplementary Figure 2 Relative enzyme activity of AGA1 and AGA2 across a range of pH values.

Supplementary Figure 3
*In situ* hybridization of *SbRS*, *SbAGA1*, and *SbAGA2* in major veins **(A-H)**, minor veins **(I-P)**, and midribs **(Q-X)** of Della leaves. Red punctate structures are transcripts. Zooming in significantly increases the visibility of the transcripts. 40X objective.
