# Supplementary figures and images for: Transcriptional regulation of the raffinose family oligosaccharides pathway in Sorghum bicolor reveals potential roles in leaf sucrose transport and stem sucrose accumulation
Source: Front Plant Sci. 2022 Dec 9;13:1062264. doi: 10.3389/fpls.2022.1062264 (PMC9785717; doi:10.3389/fpls.2022.1062264)

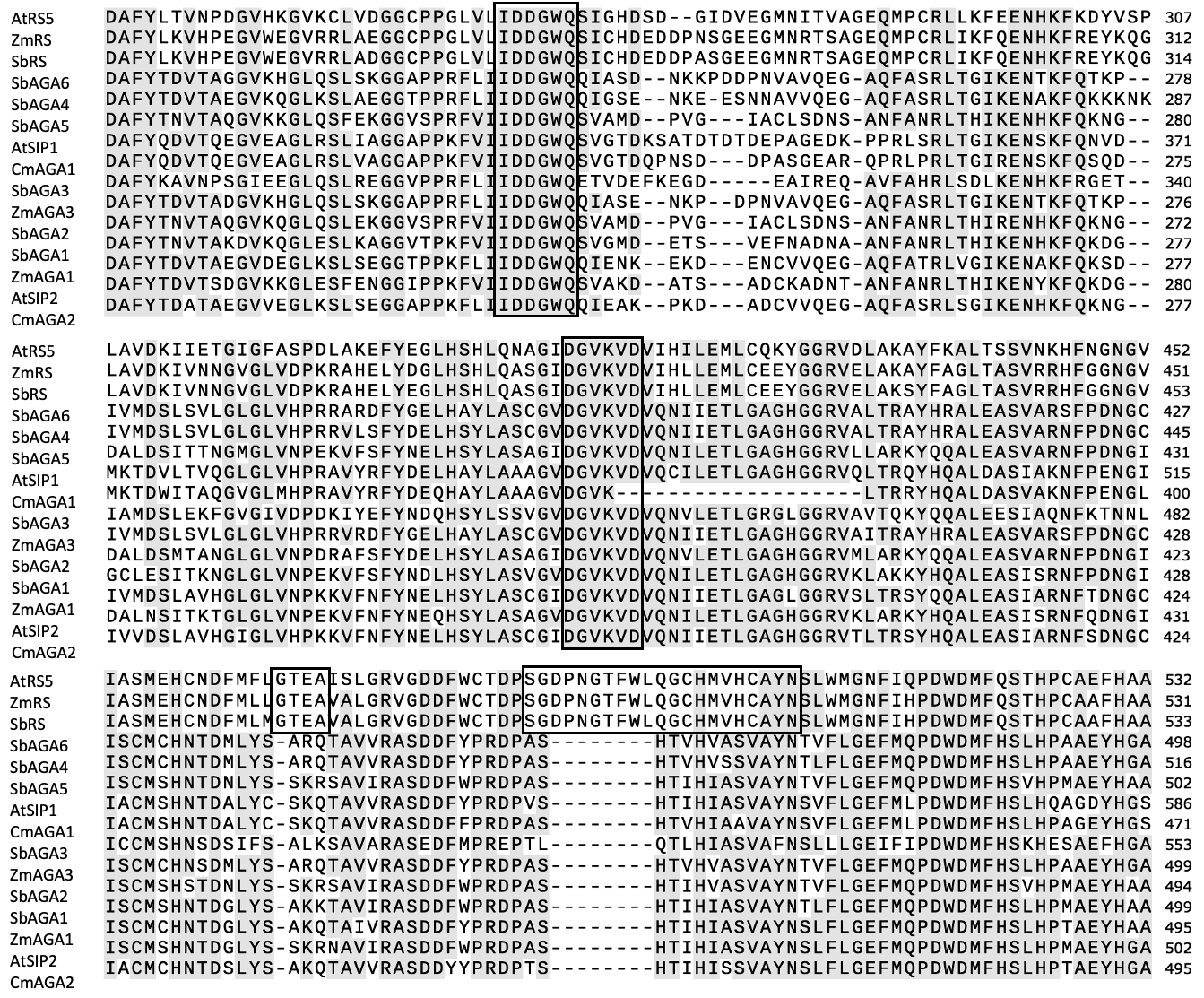

Supplement: Supplementary Figure 1 — Sequence analysis of sorghum proteins with functionally validated RS, STS, and AGA protein sequences from other species. Sequence alignments between RS and AGA proteins show sequence motifs in common [DDxW, KxD], and sequence motifs that help to differentiate RS from AGAs [FMxLGTEAxxLG, SGDPxGTxWLQGOHMVHC]. [file Image_1.tiff]

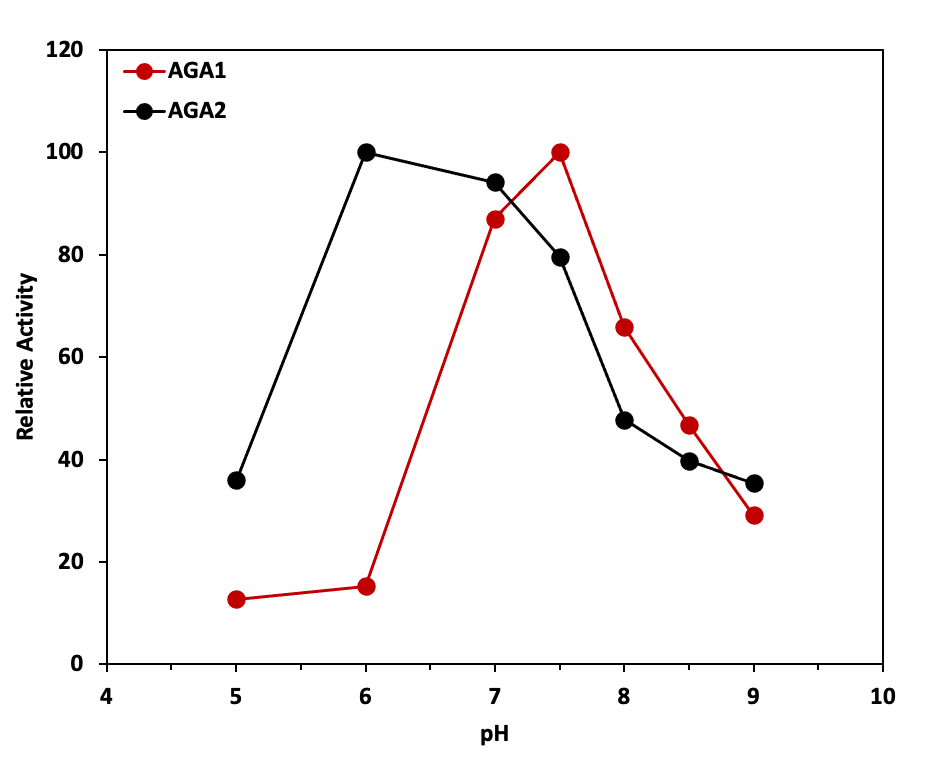

Supplement: Supplementary Figure 2 — Relative enzyme activity of AGA1 and AGA2 across a range of pH values. [file Image_2.tiff]

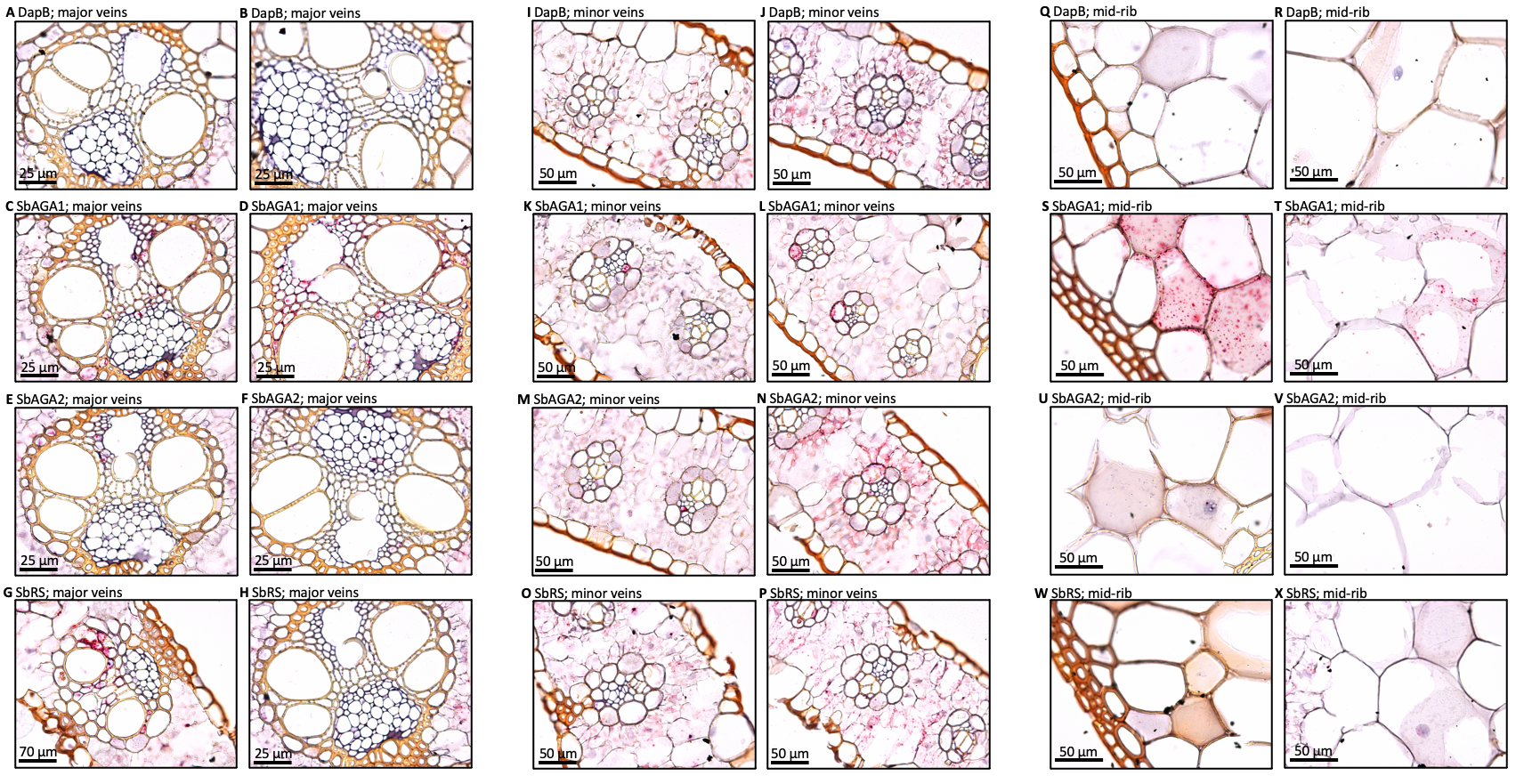

Supplement: Supplementary Figure 3 — In situ hybridization of SbRS, SbAGA1, and SbAGA2 in major veins (A-H), minor veins (I-P), and midribs (Q-X) of Della leaves. Red punctate structures are transcripts. Zooming in significantly increases the visibility of the transcripts. 40X objective. [file Image_3.tiff]
