# Supplementary material for: Transcriptional regulation of the raffinose family oligosaccharides pathway in Sorghum bicolor reveals potential roles in leaf sucrose transport and stem sucrose accumulation
Source: Front Plant Sci. 2022 Dec 9;13:1062264. doi: 10.3389/fpls.2022.1062264 (PMC9785717; doi:10.3389/fpls.2022.1062264)
Supplement: Supplementary file 4 [file DataSheet_1.docx]

**Table S1.** Gene name to gene ID conversions of genes that comprised the phylogenetic analysis. The order of the genes in the table reflects the order of genes in the phylogenetic tree.

| Gene name | Gene ID |
| --- | --- |
| VaSTS | CAB64363 |
| PsSTS | CAC38094 |
| SaSTS | CAC86963 |
| AmSTS | CAD31704 |
| AtSTS | NP_192106 |
| SbSTS | Sobic.005G210100 |
| CsRS | E15707 |
| AtRS5 | NP_198855 |
| PsRS | CAD20127 |
| OsRS | XP_015621501 |
| ZmRS | GRMZM2G150906 |
| SbRS | Sobic.003G052300 |
| ZmRS7 | GRMZM2G047292 |
| SbAGA6 | Sobic.006G122400 |
| ZmRS2 | GRMZM2G050177 |
| ZmAGA3 | GRMZM2G037265 |
| SbAGA3 | Sobic.007G219900 |
| OsAGA1 | XP_483143 |
| CmAGA1 | AAM75139 |
| LeAGA1 | AAN32954 |
| AtSIP1 | NP_175970 |
| ZmSIP2 | GRMZM2G127147 |
| SbAGA4 | Sobic.010G057300 |
| SbAGA5 | Sobic.010G057400 |
| AtSIP3 | NP_001190347 |
| AtSIP2 | NP_191311 |
| BoSIP1 | CAA55893 |
| CmAGA2 | AAM75140 |
| PaSIP1 | CAB77245 |
| ZmRS3 | GRMZM2G077181 |
| SbAGA2 | Sobic.001G044800 |
| HvSIP1 | Q40077 |
| OsSIP1 | XP_477103 |
| ZmAGA1 | GRMZM2G340656 |
| SbAGA1 | Sobic.002G075800 |
| GmAGAL1 | AAA73963 |
| CtAGAL1 | P14749 |
| AtAGAL2 | NP_001031855 |
| CaAGAL1 | Q42656 |
| LeAGAL | AAF04591 |

**Table S2.** Gene name to gene ID conversions of galactinol synthase (GolS) genes from Arabidopsis and sorghum used in this study.

| Gene name | Gene ID |
| --- | --- |
| AtGolS1 | AT2G47180 |
| AtGolS2 | AT1G56600 |
| AtGolS3 | AT1G09350 |
| AtGolS4 | AT1G60470 |
| AtGolS5 | AT5G23790 |
| AtGolS6 | AT4G26250 |
| AtGolS7 | AT1G60450 |
| SbGolS1 | Sobic.001G391300 |
| SbGolS2 | Sobic.002G423600 |
